# “Pay to Play” in the German Book Trade?

**DOI:** 10.1007/s12109-021-09804-x

**Published:** 2021-04-26

**Authors:** Corinna Norrick-Rühl, Christian Alexander Peter, Lena Schüler

**Affiliations:** 1 English Department, University of Muenster, Johannisstrasse 12-20, 48143 Münster, Germany; 2 Collaborative Research Center 1385, Law and Literature, University of Muenster, Domplatz 6, 48143 Münster, Germany

**Keywords:** Bestseller marketing, Bookselling, Brand-name authorship, German book industry

## Abstract

This paper considers the success of the thriller *Der neunte Arm des Oktopus* (transl.: *The Ninth Arm of the Octopus*, Cologne: Bastei Lübbe, November 2020) by German drugstore magnate Dirk Rossmann. Certain elements of brand-name authorship are applicable here, but the market power of Rossmann goes far beyond name recognition. Beyond the typical marketing for a ‘big book’ in the sense of contemporary trade publishing, Rossmann flooded the campaign with his own funds. This contribution approaches an unlikely case study through a trilateral interdisciplinary perspective (book studies, economics, law), underlining the unequal footing on which books enter the market.

## Introduction

At time of writing^1^ (March 2021), Dirk Rossmann^2^ rests at number 8 on the German bestseller charts for hardcover fiction books [44] with his ecocritical thriller *Der neunte Arm des Oktopus* (*The Ninth Arm of the Octopus*) [37]. 18 weeks on the *Spiegel* bestseller list (Germany’s most important bestseller list)—and counting; according to the publisher’s Spring 2021 rights guide, over 300,000 copies have been sold ([5], 9). This is an unlikely success, at least at first glance. Dirk Rossmann is not an established author, but rather a 74-year-old German billionaire and businessman who owns the drugstore chain ROSSMANN, founded in 1972.

As scholars of law, economics and book studies, we are currently working together within the collaborative research center on law and literature, funded by the German Research Foundation (DFG SFB 1385 Recht und Literatur/Law and Literature). We are all part of a project analyzing the contemporary book industry, and our interests converge in the areas of publisher branding, fixed book prices, and market dynamics. Since publication of *Der neunte Arm des Oktopus*, we have been closely following the rapid ascent of the book in the charts, and discussing it within our group. What dynamics are at play? What does Rossmann’s success say about the state of the German book industry? Where is the boundary between authorial involvement and buying one’s way into the bestseller list—or is there none?

Elsewhere, Corinna Norrick-Rühl has argued that “all novels are not created equal,” using a network metaphor to understand how “some novels are connected more firmly to central actors and institutions in the marketplace, and thus, their success is often predetermined by and through their level of networkedness” ([31], 205). To understand the success of *Der neunte Arm des Oktopus,* we will look at the different actors involved, their networkedness and their interconnections (and interdependencies).

### The Publisher: Bastei Lübbe

Bastei Lübbe, the publisher involved, is Germany’s largest non-conglomerate publisher and one of the most prolific, with a mainstay in popular fiction. Bestselling authors in their portfolio include Dan Brown, Ken Follett, Jeff Kinney and Jodi Picoult for translated books; Lübbe’s literary imprint Eichborn has authors such as Amity Gaige and Neil Gaiman on its roster. In the most recent *Global 50* report, Rüdiger Wischenbart described Lübbe as one of the few ‘major independents’ in the German market ([53], 16).

Bastei Lübbe was founded in 1953 as Bastei-Verlag Gustav H. Lübbe. The success of Bastei-Verlag Gustav H. Lübbe originally lay in the dime novel genre (‘Groschenheft’ in German; also known as ‘Heftromane’, i.e. ‘novel booklets’ in German, see [46] and [54], 441, for more context), with widely read popular series such as *Bergdoktor* (genre: medical romance) and *Jerry Cotton* (genre: crime). In 1963, the publisher expanded its offerings to include paperback books, thus entering the trade market. Today, Bastei Lübbe produces a wide range of media across ten imprints. The company went public in 2013 and, since then, the company's shares have been listed in the Prime Standard of the Frankfurt Stock Exchange (see Bastei Lübbe [3] and Wittmann [54], 450).

The publisher has, so far, come through the COVID-19 pandemic not only unscathed, but even with profits. E-book sales were a particularly successful area. Comparing the sales period April to September 2019 with April to September 2020, revenue was up 7.3% in 2020 (Bastei Lübbe [2], 2).

### The Author and His Book: Dirk Rossmann’s *Der neunte Arm des Oktopus*

Dirk Rossmann’s first foray into the book industry came in 2018, when his autobiography, co-written with two journalists *“… dann bin ich auf den Baum geklettert!” Von Aufstieg, Mut und Wandel* (“‘… I just climbed the tree!’ On moving up, courage and change”) was published by Ariston, a German Penguin Random House imprint [36]. The book received mixed reviews—as one reviewer stated “The world has not been waiting for this biography” [24]—but plenty of attention. The book achieved *Spiegel* bestseller status, even topping the list in August 2019. It certainly contributed to Dirk Rossmann’s name recognition in the wider public, and highlighted the connection between Dirk Rossmann as an entrepreneur and manager and the successful ROSSMANN brand. The autobiography and its bestseller status thus granted Dirk Rossmann the status of a brand-name author ([48], 251–252)—reinforced by his widespread name recognition through the ROSSMANN chain.

Although his drugstore chain has in the past been criticized for its lack of environmental consciousness and greenwashing (e.g. [50]), more recently he and the chain seem to be engaging more actively in a form of greenomics (cf. [52]) to improve the brand’s (and his own) reputation. In 2019, on a TV talk show, Rossmann promised on live TV to give away 25,000 copies of the German translation of Jonathan Safran Foer’s *Eating Animals* to anyone who followed the link on the ROSSMANN website [39]. Within minutes, the website had crashed [55]. According to Rossmann, this was also the point in time when he had the idea for the book *Der neunte Arm des Oktopus,* the core idea of which is a political union between the USA, Russia and China which leads to a trilateral ecological dictatorship. A sample translation of the book and brief summary in English can be found on the website of the literary agent Ute Körner, which represents the author in Spain [28].

Rossmann has openly stated that he did not write the book himself. He worked with at least a dozen people, eight of whom were researchers for the novel [18]. Nonetheless, his name acts as a brand. His chosen genre of the eco-thriller reflects on the brand for which he stands and which carries his name, and vice versa, reinforced by the fact that his book is distributed not only through traditional channels, but also in his chain of drugstores. It is the brand power behind the Rossmann name which instantly provides the book with visibility in the public sphere while the book, in turn, contributes to writing what Caroline Koegler has called the “brand narrative” (cf. [27], 118–119, 143) of the Rossmann brand. These reciprocal, performative “brand acts” channel symbolic capital in the direction of people, ideas, institutions, or indeed books. Brand acts can be considered the condition for recognizability in “the symbolic economy of discourse”, something very much emulated by the Rossmann example. As branding can go both ways, i.e. can easily turn out to be unfavorable or escape any individual’s, group’s, or institution’s control, safeguarding the brand through narratives and brand acts is something many act upon (wittingly or unwittingly), and that is arguably done here by Rossmann via the book and related publicity.^3^

### The Marketing Campaign: How Rossmann “Overran the German Book Industry with Money” (Transl. from [33], 106)

The book was launched with a 1-h virtual event hosted by Germany’s major bookstore chain, Thalia, streamed live on November 16, 2020, now available on YouTube (3804 views; status quo: March 26, 2021) [47]. Bastei Lübbe dropped the book trailer on their YouTube channel the same day (1133 views, status quo: March 26, 2021) [4]. Admittedly, these numbers aren’t particularly staggering. Nonetheless, most books do not receive this type of ‘launch pad’ on their publication day [cf. Huse [25] for context].

Bastei Lübbe has been distributing Rossmann’s book through bookstores, but the book is also stocked in more than 2000 stores of the drugstore group ROSSMANN. In the store, the book is located in an area which draws customers in: the book is nestled in between weekly rotating special offers of clothing, household electronics and other lifestyle products. Advertising boards in the entrance area in each of his stores draw attention to Rossmann’s book. During lockdown, this is particularly interesting. Besides the rack jobber’s offerings in supermarkets, Rossmann’s book is one of the few that are available to readers browsing the shelves of those few stores that are currently open.^4^

Rossmann has been giving interviews and is a welcome guest on German TV talk shows to talk about his book. As a big businessman and billionaire from Hanover, he is well-known and very well connected, his inner circle including politicians such as Christian Wulff, retired President of the Federal Republic of Germany, Gerhard Schröder, retired Chancellor of the Federal Republic of Germany and journalists such as the editor-in-chief of the weekly newspaper *Die Zeit*, Giovanni di Lorenzo.

In November last year, Rossmann appeared on the television talkshow *3 nach 9*, broadcast by multiple German public broadcasting channels (produced by Radio Bremen TV). The program was presented by di Lorenzo and the anchorwoman of the *Tagesschau*, Judith Rakers. Rossmann talked about his book for almost 20 min.^5^ He told a story—repeated often, across several media outlets—that he would always wake up at 4 a.m. thinking about the book; allegedly, he had 80% of the plot set out but did not know how best to turn it into a full-scale novel. Part of the narrative is that Rossmann sought advice from his close friend from Hanover, Christian Wulff—the aforementioned retired president of the Federal Republic of Germany. Wulff reportedly told Rossmann to talk to ‘five smart people.’ One of them was the journalist Adam Soboczynski from *Die Zeit.* Although Rossmann met with him hoping to find someone from *Die Zeit* or the magazine *Spiegel* to help him write the book, Soboczynski did not join the project. Instead, Rossmann won over his friends, the journalists Olaf Köhne und Peter Käfferlein, with whom he had co-written his autobiography the year before. With them he wrote his book, joined by other people who conducted research. In the end, at least 12 people worked on the book (cf. also [33], 107).

Rossmann told di Lorenzo during the program that the People’s Republic of China had already asked him personally whether his book could be published in China. Allegedly, a Netflix director had also contacted him. According to Rossmann, the book has already been translated into Mandarin and English. However, while the complete manuscript is available in English according to Bastei Lübbe’s foreign rights information for Spring/Summer 2021, the rights catalog does not indicate any sold rights ([5], 9). Additionally, the German national library catalog does not indicate any advance bibliographical information on expected translations, as it would if foreign publishers had already announced the book [19], status quo: March 26, 2021). The career of this book beyond the confines of the German-speaking markets is thus uncertain.

Giovanni di Lorenzo pointed out the difference between Rossmann’s book and other books: “It is advertised in ads in a format that a writer has never experienced before.” In fact, Rossmann ran (and, arguably, is still running) a far-reaching, self-financed advertising campaign. He has placed and paid for large-format advertisements for his book in all major German newspapers. But it does not stop there. He has also paid for advertising during the most coveted slot on public German TV broadcasting (ARD): immediately before the 8 p.m. news (Tagesschau). 20 s for a commercial in this time slot costs at least € 42,600 (currently approximately $51,500). Due to these high costs, TV advertising for books is atypical.

Unsurprisingly, perhaps, Rossmann’s customer magazine with the title *Centaur* included a lavishly illustrated (see [35] “review” of the book last year). As with many of these customer magazines, which are available for free in store, the print run and reach far surpasses that of most regular print magazines. The magazine is available in all German Rossmann stores, currently, in Germany alone, there are 2233 stores, with over 4000 stores across Europe [38]. In 2015, *Centaur* had a print run of one million print copies with an estimated reach of 2.5 million readers (cf. [17]); newer numbers are not available, but given the growth of the chain, the print run is probably higher by now.

### The Reception: An Inevitable Success Story? An Analysis of (Sales) Success Factors and Demand Side Dynamics

The massive advertising and media effort around the time of Rossmann’s book release quickly paid off, with the eco-thriller landing at No. 2 on the weekly *Spiegel* bestseller list (in the category ‘fiction in hardcover’) in the very first week after publication in November 2020. It has remained within the top few titles for months now. To use a British term sometimes tossed about in the book trade: Is this a case of mere ‘bungs’ (a form of bribery) ([45], 28–29) at play in the book trade, a success bought with massive advertising effort and distribution through the author’s drugstore chain? To further investigate, we take a closer look at the success factors and demand dynamics in the book market applied to the present case, which presumably have played a decisive role in making the book a bestseller.

Since books are ‘experience goods’^6^ in economic terms—i.e., the product quality or reading pleasure can only be determined after consumption has been completed—consumers often “have a hard time deciding which book to buy” ([12], 740). These pre-purchase information asymmetries—as typical for creative industries—result in substantial decision uncertainty for (potential) book buyers, usually amplified by short product life cycles and a high diversity of offerings ([26], 11–12). Due to a high degree of market saturation in the German book market, readers are exposed to a nearly unmanageable supply of books, which is—with regard to new titles—especially true for the fiction segment.^7^ Given the low level of market transparency, consumers may incur considerable (transaction) costs in their search for a novel that meets their personal preferences and requirements. To reduce the purchase risk and avoid a bad buy, readers can refer to available quality signals in the ‘market for information’ ([12], 740–741). Besides observable product characteristics such as book cover, contents and blurb, the author’s reputation, advertising measures, professional reviews in newspaper or television, bestseller rankings, or word of mouth can reduce information asymmetries on the demand side with regard to a certain book title. In this function these ‘information intermediaries’ can also represent critical success factors for a newly released book.

The massive consumer-oriented marketing measures—not financed by the publisher as is usual, but the author himself—certainly have been an important, if not the most important, factor for Rossmann’s book to become a bestselling title as they obviously surpassed the average advertising campaign (in terms of intensity as well as breadth of media use) of large publishing houses for ‘top titles’ or bestselling authors of their frontlist.^8^ While books are rarely advertised on television and radio, and placed on the feature pages of newspapers at most with small ads [49], there was, as already mentioned, a large-scale campaign (inter alia) in television, radio, and newspapers as well as the drugstore’s customer magazine for Rossmann’s book, which thus enjoyed very high public attention in Germany. Although customer advertising for books written by recent (fiction) bestselling authors seems to have, due to selection effects, only a marginal impact on sales [41], it can strongly be assumed that the intense title-specific marketing led to a far-reaching information effect on consumers with regard to (the existence of) *Der neunte Arm des Oktopus*, especially around the time of publication.

Visible in nearly every ad, prominently placed as a sticker on the book cover, and also mentioned verbatim in the TV commercial is Rossmann's status as a ‘*Spiegel* bestselling author’. This leads us to the “star power” of an author which represents an important driver of sales success for hardcover fiction titles ([14], 90–91, 96, [40], 39–42). Star power of an author can be present for two reasons. First, due to his or her fame from bestselling books in the past.^9^ Second, due to the celebrity from his or her (actual) profession, for example, on television, in sports or politics. Since Dirk Rossmann undoubtedly has the status of a bestselling (or brand-name) author due to his first commercially successful book in October 2018, the corresponding “star power” effect will have played a role in the case of *Der neunte Arm des Oktopus*. Even if the managing director of a large German drugstore chain cannot be classified as a real celebrity (i.e., the second star power effect is only weak if present at all), his name recognition may have strengthened the first star power effect on book sales. His name and label as a ‘*Spiegel* bestselling author’ will, by acting as a quality signal, have reduced uncertainties for consumers when deciding which book to buy.

His status as a bestselling author *and* public persona in turn made him a sought-after and visible author in the media, as already seen, inter alia, for numerous TV talk shows. The high media presence of Dirk Rossmann (and his new book) will have provided an additional important information and promotion effect for *Der neunte Arm des Oktopus* among potential book buyers. Another form of promotion activity that can be crucial for the first release of a book and positively influences their sales, are professional reviews on TV (see, e.g., [20] or [40]). Reviews such as Denis Scheck’s on his own TV show "Hot off the press" (transl. of “Druckfrisch”) will have further pushed the book’s popularity and, therefore, sales (particularly if it has not only an ‘information effect’ but also a ‘persuasion effect' on viewers). Professional book reviews in newspapers also have a positive impact on sales (see, e.g., [6] or [34]).^10^

The high visibility and attention in German media in particular (and strong word-of-mouth effects probably triggered by it) presumably led to the sharp increase in sales of *Der neunte Arm des Oktopus* at the time of its publication, thus explaining large parts of the high (initial) demand. As a downstream effect of high sales figures, Rossmann’s book quickly reached a top position on the *Spiegel* bestseller list shortly after its release date. This will have reinforced the market breakthrough that had already occurred, since bestseller lists and popularity rankings allow consumers to learn (indirectly) about product quality by observing other consumers’ purchase decisions ([43], 89). Empirical studies provide evidence for this “ranking effect” on book sales (cf. [15, 42]).^11^ When looking at the effect on the retail level, online sales seem to respond positively to the quality signified by a title appearing on the bestseller lists (while the offline channel shows the opposite effect) [21]. Due to the experience good character of books and the fact that the purchase (not the reading) is a relatively low-cost decision, the fiction book market is seen as a ‘social market’ where individual consumers are strongly guided by the behavior of others [26].

An important (and fortunate) circumstance for the success of the book was certainly that the entrepreneur was able to continue selling his book in his drugstore chain ROSSMANN, which has not been affected by the lockdown measures in Germany [11]. Non-book specialty stores in general (e.g., drugstores, grocery stores, or railway station bookstores) also take demand-side effects of rankings into consideration, in that they at least carry the top 10 bestselling books (which probably meet the mass taste) in their assortment. As a distribution channel they usually have a market share of less than 10% in the German book trade ([8], 8–9), with drugstores accounting for only part of this. Published sales figures indicate that the distribution of sales in the case of *Der neunte Arm des Oktopus* have been different. According to buchreport [9], about one third (i.e., more than 30%) of all sales of the book were made through ROSSMANN stores—clearly an above-average share of sales that falls on a non-book specialty store in the distribution of a book published in Germany. This illustrates the impact and relevance of pandemic and lockdown conditions, which (in addition to e-commerce) have substantially benefited secondary markets like drugstores, not only, but also as a distribution channel for books.^12^ The particularly prominent placement and high visibility of *Der neunte Arm des Oktopus* in the author’s drugstores will presumably have led to further (spontaneous) purchase effects in favor of the thriller.^13^ Dirk Rossmann and his book thus also commercially profited from the fact that the ROSSMANN drugstores were not affected by the German legislation on lockdown measures, and were among the few stores where (bestselling) books were available as well as—in the case of Rossmann’s book highly—visible in stationary form and readers were able to browse the shelves. To quote a 2020 *New York Times* piece, “If you want to sell books during a pandemic, it turns out that one of the best places to do it is within easy reach of […] diapers” [23].

However, even without other distribution channels, and without the effects of the pandemic, the probability of Rossmann’s book becoming a bestseller was fairly high. To reach the top five of the *Spiegel* bestseller list for fiction, an author requires around 9000 to 11,000 copies sold per week ([32], 36). There were times when Rossmann sold more than 2000 copies of his book per day solely through his drugstores [51], which (extrapolating it to a week) would have already secured *Der neunte Arm des Oktopus* a top position on the fiction bestseller list in Germany.

When thinking about purchased success, for (cultural) economists the term ‘payola’ comes to mind. First observed in the popular music industry (“pay to play” a song in radio channels), the phenomenon is generally defined as “a bribe paid in order to influence a gatekeeper’s choice among competing creative products” ([13], 286).^14^ Payola can also occur in the context of the book industry, with the argument running through the bestseller list. An author who is solvent by virtue of his or her actual profession could buy so many copies of his or her own work that it reaches the bestseller charts ([12], 741). From an economic point of view, this situation represents a failure of the ‘market for information’. This would have been the case if the quantity of *Der neunte Arm des Oktopus* purchased by the drugstore chain ROSSMANN (i.e., by the entrepreneur and author Rossmann) had already had an influence on the bestseller list. However, the data collection method used to compile the *Spiegel* bestseller lists should prevent this form of payola from occurring in the German book market. The market research company called Media Control is responsible for the data collection, and receives the sales figures electronically from the merchandise management systems of more than 6500 bookselling outlets on a daily basis, with drugstore chains also being included.^15^ According to the company, only actual sales are considered, i.e., book purchases must have been made by end customers.^16^ Thus, pre-orders or direct business with publishers have no direct influence on the collected data and bestseller rankings based on it. Dirk Rossmann could therefore only have ‘bought’ his way into the (*Spiegel)* bestseller lists by purchasing thousands of his books at the checkout in one of his drugstores, or other sales channels considered by Media Control, such as online booksellers (e.g., Amazon) or railway station bookstores. The present case, then, does not involve payola.

## Conclusion and Outlook

In the end, it remains to be asked how the book is actually received by the consumers. Can it convince consumers through its content and literary merit? Regardless of the market mechanisms at work, one might assume that the book would not have stayed on the bestseller lists for so long if the quality was too poor from the end consumer's point of view, or at least would be reflected in customer reviews. Granted, a close reading of the book is neither the goal of this article nor are we qualified to pass literary judgment.

As indicated, established critics ventured to review the book, with mixed results. The aforementioned Denis Scheck, a very opinionated German critic with his own TV show in which he literally throws books into a paper bin if he doesn’t approve of them, gave a measured review, said the book was written in “Scrooge McDuck prose” but still “surprisingly stimulating” (transl. from [49]). For what it’s worth, a look at the current Amazon rating of *The Ninth Arm of The Octopus* is 4.0/5 stars (status quo: March 26, 2021, 2300 ratings) [1]. Similarly, the Goodreads rating stands at 3.32/5 stars (status quo: March 26, 2021, 165 ratings) [22]. Some members of the feuilleton and traditional media have been less generous. Knut Cordsen from the Bayerischer Rundfunk slammed the book, calling it a “crude horror vision full of anti-liberal fantasies” (transl. of “krude Horrorvision voller antiliberaler Fantasien”) [16]. *Süddeutsche Zeitung’s* Rudolf Neumaier also published a scathing and satirical critique, titling it “The epiphany of Rossmann” (“Rossmanns Offenbarung”), in November 2020. Neumaier notes that while the book’s paratexts designate it as a thriller, Rossmann may have invented a new genre: the billionaire’s threepenny world rescue novel (transl. of “den Milliardärs-Dreigroschenweltrettungsroman” [30]).

The book may not be a literary masterpiece, but it is an instructive example of the unequal footing that books have upon entry into the market. Certainly, the book would not have received this level of attention (and we wouldn’t have written this article) if it hadn’t been for Rossmann’s status as a brand and his visibility far beyond the reaches of the traditional book trade—and his extraordinary financial backing of the project, which other authors cannot dream to compete with. ROSSMANN’s revenue in 2020 was 10.3 billion Euros [33], 107), in comparison, in 2019, the entire German book trade had an annual revenue of approximately 9.291 billion Euros ([8], 8). Under these conditions, German consumers had an above-average amount of quality signals at their disposal, which made *Der neunte Arm des Oktopus* a comparably low-risk (purchase) decision. It can be assumed that even without the pandemic-related competitive advantage of the ROSSMANN stores, Rossmann’s book would very likely have become a bestseller.

In lieu of a conclusion, let us add a final twist to this intricate story of ‘pay to play’ in the German book trade. In the meantime, Rossmann has founded a new company (Rossmann Media GmbH), which will receive his royalties in his stead (with company income taxed at 30% instead of the 48% income tax he would pay if he were receiving the royalties personally) ([33], 107). The icing on the cake, though, is the fact that Rossmann now owns approximately 5% of the publisher he chose to work with through his investment company (Rossmann Beteiligungsgesellschaft). Early this year, trade journals reported that Rossmann had bought shares of Bastei Lübbe. According to buchreport [9], Rossmann spent between 1.5 and 2 million Euros on 405,000 shares of Bastei Lübbe in January 2021, amounting to control of 3.05% of the voting rights of Bastei Lübbe. In April 2021, Rossmann Beteiligungsgesellschaft invested even more; he now owns 5.09%, an investment which required further clearance in accordance with the German securities trading act (Wertpapierhandelsgesetz) [7]. Arguably, the octopus is Dirk Rossmann himself.

## Footnotes

1 This research has been supported by the German Research Foundation (DFG) through our involvement in the collaborative research center SFB 1385 Law and Literature/Recht und Literatur. We would like to express our gratitude to Chandni Ananth and Mathilda Dick (both SFB 1385, Muenster) for comments and assistance with the preparation of this article. In addition, our colleague Wanda Kaufman (SFB 1385, Muenster) as well as Marcella Blum (Munich), Silvia Krysciak, Christiane Lawall and Valeria Pauz (all Berlin) proved to be valuable interlocutors during the writing of this article.

2 Dirk Roßmann’s family name uses the German special character ‘ß’, while his drugstore chain is spelled Rossmann. On the cover of his book, he has also chosen to use the spelling Rossmann. To avoid confusion, we have decided to follow the brand’s spelling throughout. When indicating the drugstore chain, we use all capitals in accordance with the registered trademark.

3 These observations were inspired by conversations with Dr. Caroline Koegler (SFB 1385, Muenster), whom we thank for her valuable input.

4 Non-essential stores, including most bookstores, were closed from mid-December 2020 to mid-March 2021. While the legislation among German federal states differed slightly, and some states permitted curbside pickup or other exceptions, book browsing was limited to supermarkets and drugstores, predominantly supplied by rack jobbers with mainstream titles.

5 In addition, there were further appearances of Rossmann in German media, e.g. in the SAT1 show “Frühstücksfernsehen” (November 25, 2020) or in the program NDR Kultur à la carte (December 21, 2020).

6 The notion goes back to Nelson [29].

7 In recent years, annual title production in Germany has amounted to just under 80,000 books; of these, 70,000 are new titles. More than 40% of new titles belong to the fiction segment ([8], 81–85).

8 For the average marketing measures taken for ‘top titles’, see the online publisher’s previews of the major German trade publishers, e.g. Random House or Bastei Lübbe, the publishing house of *Der neunte Arm des Oktopus*. The advertising consists mostly of online marketing, social media marketing, and to some extent print advertising. TV advertising is typically not used (as it is too costly).

9 In the above mentioned empirical studies, an author is granted “bestseller status” if he or she has appeared on the bestseller lists in the previous two years.

10 Since even negative professional book reviews, whether on television or in newspapers, show a positive impact on sales (cf. [6, 14], negative reviews of *Der neunte Arm des Oktopus* will potentially have increased its sales as well.

11 Besides acting as a quality signal for consumers, bestseller rankings can also increase sales as a result of (supply side) promotion activities undertaken for bestsellers [ibid., 729–730]. Due to fixed book prices in Germany, price effects (due to discounting of bestseller titles by retailers) should not occur.

12 For the year 2020 alone, *buchreport* [10] reports massive sales losses in German brick-and-mortar bookstores (minus 9%) and German railway station bookstores (minus 38%, mainly due to a passenger decline in train traffic), while online booksellers (plus 19%) and secondary markets (double-digit growth), such as grocery stores and drugstores, generated strong sales gains.

13 Furthermore, the TV commercial pointed out that the book is available in *ROSSMANN* stores, thus bringing the drugstores to the forefront as a point of sale to go to for interested readers of *Der neunte Arm des Oktopus*.

14 Since “payola runs against the vital role of objectivity (for culture) that gatekeepers perform” ([12], 741), the phenomenon has a negative connotation in the context of cultural industries.

15 This includes brick-and-mortar bookstores (independent bookstores and the chains), online booksellers, railway station bookstores, department stores, and secondary markets including consumer electronics retailers and drugstore chains with print media offerings.

16 We received the information on the considered number of bookselling outlets (German region only) and exact data collection by Media Control for the *Spiegel* bestseller list in telephone conversations with the company's book department (February 2, 2021; March 19, 2021).
